# Using Chronic Care Management Visits to Reduce the Number of Frequent Emergency Department Visits: A Pilot Study

**DOI:** 10.7759/cureus.48053

**Published:** 2023-10-31

**Authors:** Rock P Vomer, Emma York, Julian Greer, Michael S Layne, John Whitenton, Jennifer Ware, Justin F Tondt, Margaret Murray

**Affiliations:** 1 Family Medicine, Mayo Clinic, Jacksonville, USA; 2 Family Medicine, Avance Care, Raleigh, USA; 3 Family Medicine, Prisma Health University of South Carolina, Columbia, USA; 4 Family Medicine, Greer Family Practice, Ocean City, USA; 5 Family Medicine, Eastern Virginia Medical School, Norfolk, USA; 6 Emergency Medicine, ApolloMD, Henderson, USA; 7 Family Medicine, Sentara Healthcare, Norfolk, USA; 8 Family and Community Medicine, Penn State College of Medicine, Penn State Milton S. Hershey Medical Center, Hershey, USA; 9 Occupational, Environmental and Climate Medicine, University of California San Francisco, San Francisco, USA

**Keywords:** primary care, chronic care, cost, healthcare access, emergency department

## Abstract

Background

Emergency department (ED) visits are increasing in number and cost and becoming a major way patients are interacting with the healthcare system. Patients who frequently visit the ED are deemed “super-utilizers” who visit for a variety of reasons, including, but not limited to, multiple chronic medical illnesses, homelessness, and substance use disorder, but fail to have an established long-term treatment plan.

Methodology

We enrolled our hospital’s top 50 super-utilizing patients into the Chronic Care Management Program. These patients received monthly telehealth visits to discuss concerns, chronic medical conditions, barriers to care, and support systems unique to each patient’s living and social situation (i.e., social determinants of health). Telehealth visits also connected patients to community resources and helped them initiate advanced care services.

Results

The t-test investigating the frequency of avoidable visits pre- and post-intervention revealed a statistically significant decrease in the number of avoidable visits between the pre-intervention and post-intervention. Results also revealed a non-statistically significant difference in the cost of avoidable visits before and after the intervention.

Conclusions

The findings revealed a statistically significant decrease in patients’ frequency of avoidable visits before and after the intervention.

## Introduction

Emergency department (ED) visits are increasing in number and cost and are, in fact, becoming the major way in which many patients interact with the healthcare system [1,2]. Many stakeholders in patient care have tried to address this growing problem, but results to date have been varied [3]. An important remaining challenge is identifying individual patients who are frequent users, and when possible, mitigating factors that play a role in their ED presentation including coordinating appropriate outpatient management services to forestall avoidable ED visits.

A key to understanding the real-world use of ED services is the stunning fact that only 5% of the US population accounts for 25% of ED utilization [4]. Such patients are sometimes termed “super-utilizers,” an informal and possibly stigmatizing term that deflects attention from the causes of the behavior. Frequent users most often seek ED care because of homelessness, substance use disorder, and chronic medical illness [5]. Although popular perceptions often associate ED use with receipt of hospital-based social services, ED visits do not always result in the establishment of longitudinal chronic care management [6,7].

Our clinic, like many residency clinics across the United States, cares for a large population of homeless and underserved patients who have significant barriers to healthcare such as limited education and restricted financial and social resources [7,8]. These social determinants of health play a large role not only in patients’ understanding of their health and healthcare but also play a role in the way patients interact with the healthcare system [9]. Our team of family medicine residents who work both in the inpatient and outpatient settings noticed some recurring patterns, i.e., the same patients presenting frequently to the emergency room (ER) due to taking their medications incorrectly or running out of them, as well as these same patients failing to come to their appointments in the clinic following discharge. After repeatedly witnessing these patterns, we investigated solutions to help reduce our patients’ need to go to the ER for problems that can largely be treated in the outpatient setting. This led to the development of our quality improvement project creating a Chronic Care Management Team to better assist in bringing care to this patient population.

Some medical schools have outlined social determinants of health curriculum; however, there are no published resources that outline how to implement a resident-led Chronic Care Management Team [10,11]. The target audience for this resource is residency programs looking to include services for their patients other than in-person visits that will directly work to decrease ED utilization rates and expose residents to patient care scenarios where consideration of social determinants of health is integral to developing a patient care plan.

Our quality improvement project’s aim was to reduce overall ED use among the most frequent ER utilizers on the Eastern Virginia Medical School Ghent Family Medicine panel during the fiscal year. Specifically, we formed a Chronic Care Management Team of physicians and nurses who aimed to enroll the top 50 frequent users in our system. The goal of the team and the quality improvement project was to reduce avoidable ER visits among this group of individuals by addressing care needs through monthly telehealth visits.

## Materials and methods

An avoidable visit for our quality improvement project was defined as an ED visit in which the treatment plan could have been sufficiently fulfilled by our primary care office such as a medication refill. The Chronic Care Management Team identified the top 50 frequent users who had the highest number of avoidable visits during fiscal year 2020, which was obtained three months before the formation of the Team. The top 50 frequent users were identified by a claims review of the clinic’s assigned patients who presented to the emergency room. The sample size for this quality improvement study was selected based on the capacity of our team. Residents who participated in the Chronic Care Management Team were required to have knowledge of the management of chronic medical diseases, the ability to triage on the phone for appropriate utilization in various healthcare settings, and knowledge of community resources that patients may benefit from.

Patients who elected to enroll received a monthly phone or video telehealth visit from one or more members of the Chronic Care Management Team to assess chronic medical conditions, identify any barriers to care (in the context of the social determinants of health), address specific patient concerns, and coordinate needed services as appropriate. Each patient was contacted monthly via phone using a scripted phone message. Patients were also provided written educational materials outlining chronic care visits.

Seven patients verbally consented to receiving monthly Chronic Care Management phone calls. Additional identified frequent ER-using patients either declined to enroll or were unable to be contacted. During each successful phone visit with the seven patients, patients were encouraged to contact the Ghent Family Medicine office number or after-hours line before presenting to the ED outside of their monthly Chronic Care Management visits or other routinely scheduled office visits. To identify gaps in care, the team was also alerted when any patient presented to the ED while enrolled in the Chronic Care Management program.

Based on the number of enrolled patients, the four participating residents were assigned one or two patients and called them monthly for three months. First-year residents were assigned one patient, while senior residents were assigned two patients. During the first month, patients and providers also identified convenient phone call times for future visits. Each of the three phone calls addressed the patient’s concerns, chronic medical conditions, barriers and support systems unique to each patient’s living situation, and any social determinants of health, including connecting patients to community resources and initiating Advance Care Planning conversations [12,13]. Social needs were determined using the American Academy of Family Physicians Social needs tool [14]. Each Chronic Care Management visit followed a structured template, and the visit was recorded as a patient correspondence note in our electronic medical record system, Allscripts. Each Chronic Care Management patient had their own individualized healthcare plan, and visits were billed using the clinical staff charge of 99490. The number of subsequent ED visits was recorded for three months, using an Excel sheet that excluded patient-identifying information, to determine if there was a reduction in ED presentations after the patient was enrolled in chronic care management.

## Results

The independent variable (IV) time comprised two levels (1 = before the intervention, and 2 = after the intervention). The dependent variables (DVs) included the frequency of avoidable visits and the cost of visits. Two separate paired-sample t-tests were computed instead of a one-way multivariate analysis of variance due to statistical power considerations (N = 6). The following statistical assumptions and requirements for a paired-sample t-test provided by Rovai et al. (2013) were checked: one categorical level IV with two levels and one continuous level DV, data were dependent, and the sampling distribution of differences between scores was normally distributed. The results of both the Shapiro-Wilk and Kolmogorov-Smirnov tests of normality revealed that the sampling distribution of differences between scores was consistent with a normal distribution (p > 0.05).

The first paired-sample t-test was computed to investigate differences in the frequency of avoidable visits between the pre-intervention and post-intervention. Results revealed a statistically significant decrease in the number of avoidable visits between the pre-intervention (mean = 6.83, SD = 2.79) and the post-intervention (mean = 3.00, SD = 2.00) (95% confidence interval (CI) = 0.91, 6.76, t(5) = 3.37, p = 0.020, d = 1.58) (Table 1). The second paired-sample t-test was computed to investigate differences in the cost of avoidable visits between the pre-intervention and post-intervention. Results revealed a non-statistically significant difference in the cost of avoidable visits between the pre-intervention (mean = 5436.19, SD = 3972.82) and the post-intervention (mean = 2187.19, SD = 2160.74) (95% CI = -1668.90, 8166.89), t(5) = 1.70, p = 0.150, d = 1.02).

**Table 1:** Number of visits pre and post-intervention. This table demonstrates the number of emergency room presentations before and after the chronic care management intervention.

	Pre-intervention			Post-intervention		
Subject number	Visits	Type	Cost	Visit	Type	Cost
1	1/23/2019	Avoidable	$163	10/26/2020	Avoidable	$303
	2/9/2019	Emergent	$1,742	1/1/2021	Emergent	$625
	3/8/2019	Other	$510	1/20/2021	Avoidable	$97
	3/17/2019	Other	$263			
	4/30/2019	Avoidable	$826			
	5/17/2019	Other	$93			
	6/17/2019	Avoidable	$566			
	8/2/2019	Other	$356			
	9/3/2019	Avoidable	$455			
	9/4/2019	Other	$548			
	1/30/2020	Other	$488			
	2/22/2020	Other	$1,420			
	3/17/2020	Other	$163			
	9/14/2020	Emergent	$615			
2	2/6/2018	Other	$346	12/19/2020	Avoidable	$1,104
	2/12/2018	Other	$483			
	6/12/2018	Other	$1,729			
	11/24/2018	Avoidable	$5,586			
	3/9/2019	Avoidable	$309			
	7/18/2019	Avoidable	$566			
	12/10/2019	Avoidable	$163			
	12/26/2019	Avoidable	$383			
	12/29/2019	Avoidable	$394			
3	3/8/2019	Emergent	$263	10/11/2020	Other	$571
	4/1/2019	Other	$17,026	10/19/2020	Other	$6,239
	5/8/2019	Avoidable	$1,880	11/2/2020	Other	$934
	6/19/2019	Avoidable	$511	11/24/2020	Emergent	$321
	7/27/2019	Other	$17,014	11/27/2020	Other	$1,376
	1/14/2020	Avoidable	$249	12/4/2020	Avoidable	$321
	2/11/2020	Other	$9,171	12/28/2020	Other	$910
	2/24/2020	Avoidable	$516	1/4/2021	Other	$6,279
	2/26/2020	Avoidable	$1,135	1/26/2021	Avoidable	$955
	4/7/2020	Avoidable	$358	2/1/2021	Other	$1,098
	4/10/2020	Avoidable	$306	3/2/2021	Emergent	$4,908
	4/13/2020	Emergent	$10,455	3/27/2021	Avoidable	$803
	4/22/2020	Avoidable	$6,881	3/29/2021	Avoidable	$990
	5/18/2020	Other	$7,642			
	7/1/2020	Other	$6,224			
	7/25/2020	Other	$10,784			
	8/18/2020	Avoidable	$2,126			
	8/25/2020	Other	$1,623			
	9/22/2020	Other	$5,726			
4	1/15/2019	Avoidable	$566	11/8/2020	Avoidable	$795
	1/18/2019	Other	$473	11/9/2020	Avoidable	$225
	2/3/2019	Other	$410	11/11/2020	Emergent	$398
	2/12/2019	Other	$263	11/16/2020	Other	$434
	2/18/2019	Avoidable	$575	11/19/2020	Avoidable	$542
	3/4/2019	Avoidable	$566	11/22/2020	Emergent	$6,393
	3/26/2019	Other	$593	12/11/2020	Emergent	$7,617
	3/30/2019	Avoidable	$511	1/8/2021	Other	$10,075
	4/5/2019	Emergent	$5,394	1/19/2021	Other	$987
	4/16/2019	Emergent	$429	1/26/2021	Other	$596
	4/17/2019	Avoidable	$429	2/7/2021	Other	$10,385
	5/3/2019	Avoidable	$345	2/28/2021	Avoidable	$480
	5/19/2019	Emergent	$520	3/6/2021	Emergent	$556
	5/28/2019	Emergent	$263			
	6/28/2019	Emergent	$2,888			
	7/31/2019	Other	$737			
	8/1/2019	Other	$163			
	8/21/2019	Other	$9,247			
	9/13/2019	Avoidable	$429			
	11/5/2019	Other	$701			
	11/24/2019	Other	$93			
	11/29/2019	Avoidable	$545			
	1/1/2020	Avoidable	$256			
	1/24/2020	Avoidable	$158			
	2/27/2020	Emergent	$10,045			
	5/19/2020	Other	$9,612			
	6/5/2020	Other	$255			
	6/29/2020	Other	$434			
	7/4/2020	Emergent	$434			
	8/8/2020	Avoidable	$614			
	9/1/2020	Other	$8,185			
5	1/2/2019	Avoidable	$339	12/16/2020	Avoidable	$571
	1/6/2019	Avoidable	$263	12/21/2020	Avoidable	$4,279
	1/12/2019	Other	$263	12/28/2020	Avoidable	$321
	1/22/2019	Other	$263	1/4/2021	Other	$5,618
	6/6/2019	Other	$163	3/12/2021	Avoidable	$458
	6/13/2019	Avoidable	$163	3/16/2021	Other	$276
	6/14/2019	Other	$261	3/17/2021	Other	$176
	6/19/2019	Other	$263	3/20/2021	Other	$276
		Avoidable	$163	3/22/2021	Other	$345
	6/25/2019	Avoidable	$163	3/23/2021	Avoidable	$176
	6/28/2019	Other	$163	3/26/2021	Avoidable	$269
	7/2/2019	Avoidable	$163		Avoidable	$176
	12/11/2019	Avoidable	$241	3/28/2021	Other	$458
	12/18/2019	Other	$149			
	12/23/2019	Avoidable	$149			
	12/30/2019	Other	$241			
	1/14/2020	Avoidable	$158			
	1/24/2020	Avoidable	$158			
	5/20/2020	Avoidable	$788			
6	2/15/2018	Other	$447	11/4/2020	Avoidable	$435
	2/21/2018	Other	$383			
	4/21/2018	Avoidable	$429			
	6/30/2018	Avoidable	$207			
	12/8/2018	Emergent	$429			
	1/26/2019	Avoidable	$466			
	4/5/2019	Other	$263			
	4/28/2019	Avoidable	$163			
	5/5/2019	Avoidable	$309			
	6/16/2019	Avoidable	$566			
	6/26/2019	Other	$755			
	7/30/2019	Other	$8,141			
	8/31/2019	Avoidable	$1,082			
	10/16/2019	Avoidable	$736			
	10/30/2019	Avoidable	$3,031			
	11/29/2019	Avoidable	$0			
		Avoidable	$528			
	9/13/2020	Avoidable	$434			

## Discussion

The findings revealed a statistically significant decrease in patients’ frequency of avoidable visits before and after the intervention. The effect size estimate was in the large range based on the guidelines for interpreting Cohen’s d provided by Sink and Mvududu [10]. This suggests that patients’ participation in the intervention was associated with a notable reduction in their frequency of avoidable visits. To this end, the visible mean differences in cost between the pre-intervention and post-intervention should be interpreted tentatively, if at all. However, the estimate of effect size (Cohen’s d) should not be ignored, as it was in the large range according to Sink and Mvududu [10]. Specifically, the cost of avoidable visits before the intervention was 1.02 standard deviations higher than the cost of avoidable visits after the intervention. It is possible that this mean difference occurred by random chance. However, it is also possible that the non-significant p-value that emerged in the present study was due to the low sample size. A retrospective power estimate revealed that with N = 6, there was only a 28% chance of finding statistically significant differences if statistically significant differences exist. Future researchers should test the differences in the cost of avoidable visits before and after the intervention using a larger sample.

Given the above results and the ability of the Chronic Care Management team to improve patient care, this is a reasonable addition to residency clinics. By giving residents the opportunity to have frequent contact with their assigned patients in the Chronic Care Management model, they gain a greater appreciation for patient care in the context of social determinants of health as the super-utilizer population is at greater risk of being adversely affected by these common issues. In the context of primary care, the Chronic Care Management team provides significantly increased continuity of care in a manner that our patients who were enrolled in Chronic Care Management were receptive to. Additionally, with the increased use of Telehealth services nationwide, the services provided to patients in the Chronic Care Management model can be billed for whether they are performed as phone or video encounters.

As not all super-utilizer patients were open to the idea of monthly phone calls, not all of our 50 super-utilizers were able to be enrolled in the program. Finding a way to bring Chronic Care Management services to additional at-risk patients would be helpful for the continued development of Chronic Care Management. Additionally, patients must have reliable access to a phone they can use to be enrolled in the program. One limitation of this resource is the reliance on residents to perform the phone calls for Chronic Care Management services which adds to their overall workload. In contrast, these could be either scheduled telephone visits during the resident’s clinic blocks, if the normal clinic hours work for the patients to be contacted during, or phone calls could be made by the office nursing staff and residents notified if additional physician-patient contact is needed.

For our patient population, this proved to be a successful intervention for improving patient care. We hope to continue the Chronic Care Management Team to provide better patient access to primary care resources and decrease the number of unnecessary ED visits our patients utilize. We hope to continue to refine our Chronic Care Management methods to enroll more patients and involve more residents.

Through our quality improvement project, we learned a Chronic Care Management Team can be successfully established at a residency clinic and can bring additional needed resources to an oftentimes underserved population.

## Conclusions

Though visits to the ED are increasing in frequency, quality improvement programs such as Eastern Virginia Medical School’s Chronic Care Management Program can help reduce the frequency of avoidable visits. To do this effectively, it is imperative for attending physicians, residents, and nurses to consider social determinants of health when evaluating patients who visit the ED for problems that can be managed in the outpatient setting. Enhanced resident education surrounding social factors impacting patient care can help make preventative telehealth visits/monthly phone calls more effective for patients in need of assessments of chronic medical conditions and evaluations of other specific medical concerns. In essence, the Chronic Care Management Program helps shed light on the importance of identifying significant barriers to healthcare such as limited education and restricted financial and social resources in patients. This ultimately helps make patient-centered continuity of care accessible to populations characterized as “super-utilizers” or patients who have suffered from gaps in care.
